# Rare Intracardiac Tumor: Primary Cardiac Lymphoma Presenting as Atypical Angina

**DOI:** 10.1155/2016/4125295

**Published:** 2016-01-19

**Authors:** Karthigesu Aimanan, K. Saravana Kumar, M. N. Mohd Arif, Md Noor Zuraini, Md Jamil Ramdzan, L. Hamdan

**Affiliations:** ^1^Department of General Surgery, National University of Malaysia, Cheras, 56000 Kuala Lumpur, Malaysia; ^2^Department of Cardiothoracic Surgery, Serdang Hospital, 43400 Selangor, Malaysia; ^3^Department of Cardiothoracic Anesthesia, Serdang Hospital, 43400 Selangor, Malaysia

## Abstract

Primary lymphomas of the heart are extremely rare, accounting for 2% of all primary cardiac tumors. Due to the rare presentation, there is no proper consensus available on treatment strategy. Preoperative confirmation of the pathology is fundamental in guiding an early treatment plan, which allows for improved prognosis. Unfortunately, in most cases, primary cardiac lymphoma is only identified on postoperative histopathological analyses, which affect the treatment plan and outcome. Here, we report a unique case of primary cardiac lymphoma presented with dyspnea and reduced effort tolerance. Young age, rapid onset of symptom, and absence of cardiac risk factors prompted us towards further imaging and emergency resection. The patient received a course of postoperative chemotherapy and was disease-free on six months of follow-up.

## 1. Background

Primary cardiac lymphoma is a very rare malignancy, which is typical of a non-Hodgkin's type and involves only the heart and pericardium with no or minimal evidence of extracardiac involvement. It accounts for ~1% of the primary cardiac tumors and ~0.5% of extranodal lymphomas [1]. Both B- and T-cell lymphomas have been reported. The right atrium and right ventricle are the two most frequently involved sites. Clinical presentation is heterogeneous and is generally related to the site of involvement in the heart.

## 2. Case Report

**Figure 1:**
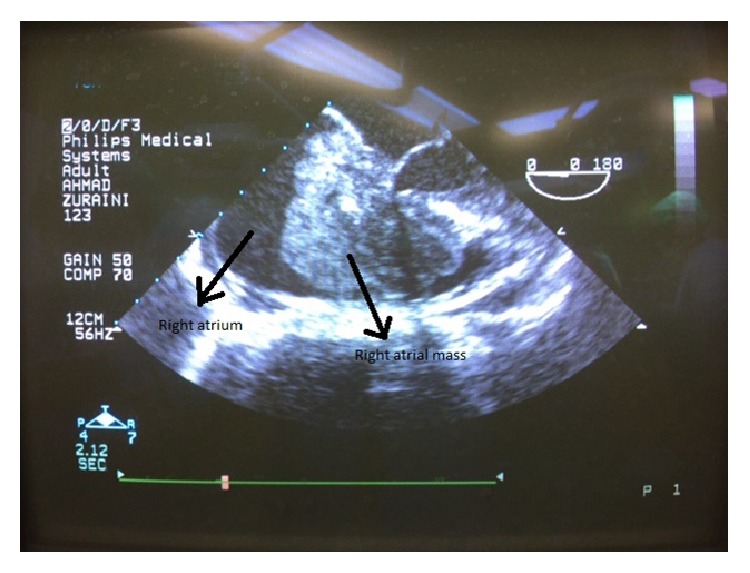
Intraop. transechocardiograph shows right atrial mass attached to interatrial septum.

**Figure 2:**
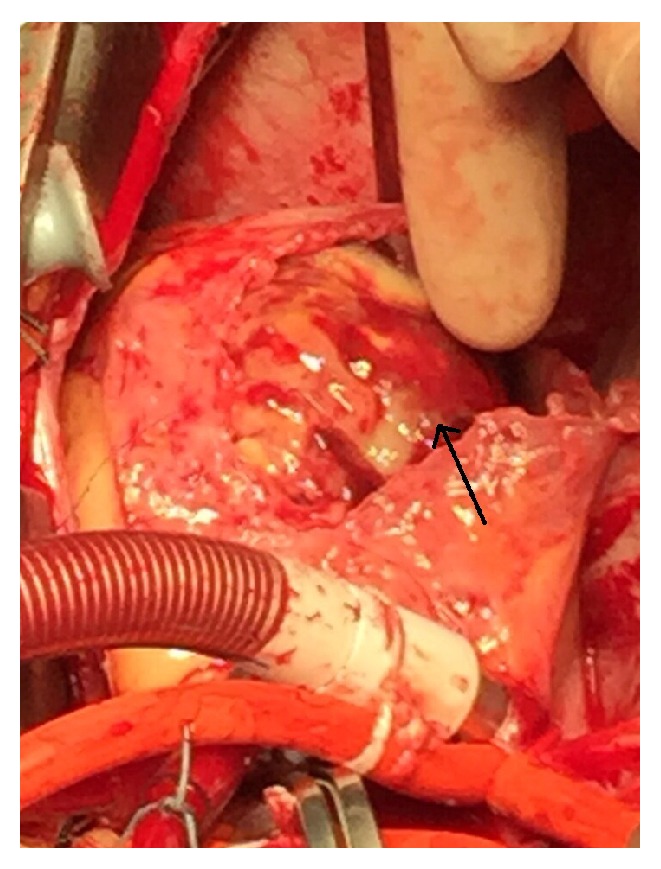
Intracardiac tumor approached through right atriotomy.

A 44-year-old female lecturer presented with 1-week history of reduced effort tolerance and intermittent central chest pain. Otherwise, she denied fever, upper respiratory tract infection symptoms, or any other medical illnesses. On examination, she was tachypneic with stable vital signs. Her ECG showed sinus rhythm with T wave inversion at V2–V5 leads. Intermittently, she had paroxysmal atrial fibrillation that aborted spontaneously. Chest X-ray revealed no features of heart failure such as cardiomegaly, Kerley B line, or pleural effusion. She was diagnosed as non-ST elevated myocardial infarction and admitted to the cardiology ward. Transthoracic echocardiography was done and showed a large lobulated mass, measuring 10 cm in the right atrium and obstructing the right ventricular outflow tract. Urgent magnetic resonance imaging revealed a soft tissue lesion within the right atrium measuring about 5.4 × 3.2 cm × 5.0 cm. It was attached to the interatrial septum and prolapsed through and obstructs the tricuspid valve into the right ventricle. The lesion abuts the left ventricular outflow tract and the aortic annulus. This case was discussed in the multidisciplinary meeting and decided for emergency surgery. Induction of anesthesia was carried out carefully. After initiation of cardiopulmonary bypass, the mass was approached through right atriotomy. A large firm, smooth, and lobulated mass was identified at the right atrium impinging the tricuspid valve and prolapsing into the right ventricle (Figure 1). The mass has a broad base, attached to the interatrial septum at the area of AV node region. The mass was excised and shaved from the interatrial septum. On palpation, there was thickening of interatrial septum just above the right ventricle. Transesophageal echocardiogram confirmed the finding (Figure 2). However, we did not resect interatrial septum due to the concern of complete heart block later. After resection of the mass, tricuspid annuloplasty was performed using Prolene 6-0 to oppose all the three segments of tricuspid leaflets. She was hemodynamically stable throughout the surgery and was extubated the next day. During follow-up, the patient did not have any failure symptoms. However, her histopathology result revealed diffuse large B-cell lymphoma. The tumor margin was positive near the interatrial septum. She was subsequently referred to oncology team and commenced on Rituximab-CHOP chemotherapy. Her follow-up scan at six months showed no local recurrence or metastasis.

## 3. Discussion

Primary cardiac tumors are rare, with a postmortem incidence of 0.05% [2]. Cardiac involvement of a systemic lymphoma has been reported in up to 20% of cases, but primary lymphoma with the bulk of the tumor involving the heart is very rare, accounting for 1% of primary cardiac tumors. Diffuse large B-cell lymphoma is the most common histologic type of cardiac lymphoma in the literature. High level of suspicion is necessary for primary cardiac lymphomas because unlike most other cardiac tumors it is rapidly progressive and fatal soon after diagnosis. Clinical presentation varies and depends on location, size, and growth rate and thus leads to delay in diagnosis. Young age, rapid onset of symptoms, and absence of cardiac risk factors were the factors against our initial diagnosis of an acute coronary syndrome that prompted us towards early imaging investigations. Among the diagnostic tools, a transthoracic echocardiogram is the most easily available and useful tool to identify at least the presence of cardiac mass. Its sensitivity depends on the size of the mass and the acoustic window of each patient. A transesophageal echocardiogram is a better alternative in terms of accuracy. It includes improved resolution of the tumor and its attachment, the ability to detect some masses not visualized by TTE, and improved visualization of right atrial tumors [3]. Further imaging with computed tomography and magnetic resonance imaging not only allow the diagnosis of infiltrating masses but also provide information about the type of tumor, vascularization and necrosis [4]. Recent advances in PET scan give us an option for further ascertaining nature of the intracardiac mass. A retrospective case series has reported that 18F-FDG PET can be used to determine tumor malignancies with a sensitivity of 100% and specificity of 86%. However, there are no much series available to further support this data, so the careful patient selection is important for a better cost benefit.

Obtaining histopathology before surgery is ultimate for further decision making. However, it is not routinely practiced for intracardiac tumors. Transvenous biopsy is a technique described in the literature for intracardiac tumors. We did not employ this technique in this patient due to the failure to anticipate malignant nature of the mass due to young age and immunocompetent adult. Besides that, transvenous biopsy technique also has a risk of dissemination of embolus resulting in pulmonary embolism. Pericardial fluid cytology has been described in cases where the mass involves pericardium [3].

Definitive treatment of primary cardiac lymphoma is still not established. Emergency surgical resection followed by chemotherapy has become the standard practice in most centers possibly due to life-threatening hemodynamic compromise on presentation. Primary cardiac lymphoma, unlike other cardiac malignancies, responds well to chemotherapy. The addition of monoclonal antibody, Rituximab, to the standard regime CHOP (cyclophosphamide, doxorubicin, vincristine, and prednisolone) has shown good outcome in tumors with positive reactivity to CD20 [4]. Radiotherapy is another modality in treating primary cardiac lymphoma but only applicable in patients with good hemodynamics or diffuse disease. In this patient, emergency surgery is indicated because of unstable hemodynamics on presentation. Nascimento et al. in their case series showed that patients who were treated with a combined modality of systemic chemotherapy and additional postchemotherapeutic radiation therapy had a better long-term progression-free survival compared to those treated with systemic chemotherapy alone [5].

## 4. Conclusion

Intracardiac mass has various complex pathologies, and cardiac lymphoma should always be ruled out even though it is a rare cause. Identification of the patients' initial presentation is essential for the subsequent management plan. PET scan should be included as early as possible in investigations for better functional analysis of the lesion. Generally, systemic chemotherapy followed by radiotherapy has shown a good outcome for primary cardiac lymphoma. However, in patients with unstable hemodynamic status, emergency resection followed by chemotherapy remains a better alternative.
